# Conceptualizing mental health stigma in organizational settings: a sociolinguistic perspective

**DOI:** 10.1186/s40359-024-02127-4

**Published:** 2024-11-30

**Authors:** Jasper Zhao Zhen Wu, Olga Zayts-Spence, Zoë Fortune

**Affiliations:** 1 Department of English, Linguistics and Theatre Studies, National University of Singapore, 7 Arts Link, Block AS5, Singapore, 117570 Singapore; 2 School of English, The University of Hong Kong, Hong Kong SAR, People’s Republic of China; 3 School of Social Sciences, Heriot-Watt University Dubai, Dubai, United Arab Emirates

**Keywords:** Stigma, Mental health, Discursive space, Organizational setting, Hong Kong

## Abstract

**Background:**

Sociolinguistic research on workplace mental health stigma is scarce and consequently, there are a lack of relevant conceptual models. Drawing on Goffman’s notion of stigma as a ‘language of relationships’, and Heller’s concept of ‘discursive space’, this paper offers a conceptual model of how stigma is produced and reinforced in workplace settings. Specifically, the model maps the complex discursive processes of mental health stigmatization through workplace discursive practices.

**Methods:**

The model is empirically grounded and draws on 23 in-depth participant interviews with professional services employees in Hong Kong. Through a meta-discursive analysis of the employees’ experience in the workplace, the paper investigates how mental health stigma is produced in the workplace.

**Results:**

Conceiving the workplace as a discursive space, the model demonstrates that mental health stigma unfolds across three discursive layers, namely immediate encounters, organizational practices, and societal ideologies. Mediated by discursive practices, such as identity management, stigma is both produced and perpetuated across the three layers.

**Conclusions:**

The paper provides a model for analyzing the production of mental health stigma through dynamic discursive activities in the workplace. By doing so, it offers a way to systematically map how stigma, brought about through discourse in organizational settings, can regulate both interpersonal relationships and resource allocation (such as career prospects).

## Introduction

The recent COVID-19 pandemic has been a catalyst for increased attention on mental health. The “parallel epidemic” of deteriorating mental health according to the World Health Organization [1] has meant that both academic and non-academic research on mental health has burgeoned since the start of the pandemic. This has been accompanied by an increasing number of practical initiatives to improve mental health.

There has also been growing attention to mental health in the field of sociolinguistics, [2, 3] with specific attention on stigma. In sociolinguistics, stigma has been studied in relation to language differences, class distinctions, gender and sexuality, and physical illnesses [4–7]. But mental health stigma remains an under-explored area in the field [8, 9] and primarily studied in psychology and sociology [10–13]. However, while sociological research has recognized the multi-layered construction of mental health stigma, sociolinguistics has much to offer in unpacking the processes of stigmatization by which alienation and inequality are perpetuated [14–16]. This concerns both symbolic processes, such as labelling individuals with mental illness as ‘failures’, and material processes, such as the limiting of promotion and employment opportunities [10, 11, 17].

This paper examines discourses of mental health in one of the key social domains – the workplace. The workplace is, arguably, the place where most people (at least in an urban society) spend most of their time. This is especially true as ‘working from home’/ hybrid working has become a common practice during and after the pandemic. While our study does not focus on this practice per se, it highlights an aspect of the workplace that is increasingly documented and examined: that the workplace is a network of sites extending beyond the traditional office. Previous sociolinguistic studies of workplaces have not focused extensively on mental health stigma nor this new, extended nature of the workplace [18–20].^.^

The paper develops a conceptual model of the discursive production of mental health stigma in organizational settings. The model is empirically grounded in a meta-discursive analysis of narratives of workplace experiences in Hong Kong. Our analysis brings together different strands of research on stigma, discursive spaces, and identity management [14, 21, 22]. We address two questions: how is mental health stigma discursively produced? And how does it influence both interpersonal relationships and resource allocation in the workplace? The interview data are drawn from our workplace mental health study spanning over five years (2018–23), encompassing the periods pre-, during- and post-pandemic. Situating the study in the context of Hong Kong allows us to examine mental health stigma beyond the dominant research settings in this area of the Global North and West. The city’s deteriorating mental health is also reflective of the global trend [23], offering an appropriate setting for the study.

We begin with a brief account on the mental health landscape and the sociocultural context that have contributed to shaping mental health stigma in Hong Kong. This is followed by our theoretical framework – stigma as a language of relationships, the workplace as a discursive space, and identity management as a discursive practice. Based on the framework, we analyse the development of mental health stigma across three discursive layers: immediate encounters, organizational practices, and societal ideologies. We then introduce the Butterfly Model of Mental Health Stigma in Organizational Settings derived from the analysis, and discuss the conceptual and practical value of the model for sociolinguistics and anti-stigma research.

## Background: mental health and stigma in Hong Kong

In 2010, it was estimated that approximately 13.3% of adults from the ethnic majority (i.e., Chinese) had experienced common mental disorders [24]. Since that time, the mental health and well-being of the population has been declining and has also been impacted by a number of environmental shocks including SARS, Covid and the recent social unrest with high numbers of the population estimated to have experienced symptoms relating to anxiety, depression and Post-Traumatic Stress Disorder [25].

There are also notable concerns amongst the workplace with a significant proportion of the workforce reporting symptoms of anxiety (31%) and depression (24%) [26]. Associated factors include long working hours (42 h per week on average [27]) [28] as well as the persistence of mental health stigma [29–34].

Furthermore, in the workplace, concealment of mental health problems is common due to the anticipation of being stigmatized [32, 35, 36]. For example, studies estimate that only around 30% of workplace members in Hong Kong have told someone at work about their condition [37]. The situation is compounded by a lack of appropriate resources with an estimated 90% of employees considering support offered by firms as sufficient [37]. Employment opportunities for individuals with mental health problems are also significantly limited by stigma [38]. Reasons for the development and persistence of stigma include inaccurate or insufficient knowledge about mental health issues (known as mental health literacy) [39, 40] although research has also cautioned against the assumption of a simple positive relation between mental health literacy and de-stigmatization [30]. Instead, studies have also pointed to the influence of traditional Chinese cultural values (such as self-restrain and face-saving) which can play a role in the perpetuation of mental health stigma [34, 41–43].

Existing interactional research has discussed the experience of anticipated stigma at work, and also presented a range of factors contributing to the perpetuation of mental health stigma. However, how and in what way these factors interact in the production of mental health stigma in the workplace remain under-explored. Broader literature in sociology has examined the multi-layered structure of mental health stigma in the workplace, but it has focused primarily on Global North and West [16]. There is, therefore, a notable gap in the conceptualization of stigma production and maintenance in the workplace. This paper aims to address this gap, focusing on workplace mental health stigma in Hong Kong [44].

## Theoretical basis for study

### Stigma as a language of relationships

Goffman’s commonly cited definition of stigma is ‘an attribute that is deeply discrediting’ [12, 13, 21]. However, often overlooked within this definition is Goffman’s view that “a language of relationships, not attributes, is really needed” for stigma to be produced [21] (p3). Attributes are ascribed to traits that might be discrediting (visible, such as a lost limb) or discreditable (not immediately visible, such as depression). These attributes are seen as markers of difference, exploited in discursive practices such as labelling, stereotyping, othering and devaluation [12]. This constitutes the ‘language of relationships’ differentiating ‘the deviant’ from ‘the normal’ [21].

Pescosolido and Martin suggested that stigma can be experienced in different ways. Stigma could be induced by others in generalized beliefs (perceived stigma) or overt behaviours of rejection (received stigma) [13]. It could also be invoked by the self through the internalization and endorsement of existing stereotypes (endorsed stigma). However, in between the self and the other, stigma can also be experienced in the expectation of prejudice and discrimination (anticipated stigma). For instance, an employee may disguise their mental illness as a physical health issue (e.g., a headache), in anticipation that speaking about their mental health problem will be detrimental to their career. In this case, there are neither stereotypes endorsed by the individual nor realized acts of devaluation performed by others.

Regardless of the form of enactment or experience, stigma is said to produce relationships of difference, alienating the stigmatized individuals from the rest of society. This constitutes a system of knowledge by which people with mental illness are discursively made into stigmatized individuals (‘subjectification’) [45]. However, it is crucial to note that this system of knowledge is not merely symbolic but also has a material dimension, as we explain below.

### The workplace as discursive space

Discursive spaces, as defined by Heller, “assemblages of interconnected sites, […] traversed by the *trajectories* of participants and of *resources* regulated there” (emphasis original) [14] (p11). The workplace constitutes both a *symbolic system* regulating interpersonal relationships (how individuals are categorized and positioned), as well as a *material system,* regulating the allocation of resources (access to capital and opportunities) [10, 11]. Stigma against persons with mental illness is articulated in a language of relationships, categorizing them as dangerous and less trustworthy, and alienating them from the ‘normal’ workforce. The concept of discursive space thus explores the dual structure of symbolic and material gatekeeping practices in the workplace.

A discursive space is a network of participants and resources. This expands the established sociolinguistic conceptualization of the workplace as an enclosing contextual backdrop [18]. With regards to mental health stigma, for instance, the decision to reject an applicant with a mental health problem might be motivated by the company’s structural practices or societal stereotypes against mental illness influenced by media representations [11, 46, 47]. The concept of discursive space emphasizes the ‘trajectories’ that extend beyond the immediate site of interaction. This allows us to reveal how stigmatizing practices in other social domains influence the allocation of resources in the workplace [14] (p11).

Stigma, however, is not necessarily enacted overtly. Its regulative force is observable notably in discursive practices of identity presentation—by the self and the other [21]. This involves both the performative disclosure and concealment of one’s mental ill health. Such practices of identity management therefore also affect an individual’s interpersonal relationships and access to resources in the workplace.

### Identity management as discursive practice

Identity is always relational, produced between the self and the other [22]. It is a matter of presentation – how one self-presents and how one is presented by others [21, 48]. Practices of presentation are achieved through semiotic (i.e., linguistic and extra-linguistic) means, with the aim to claim, attribute, reject, or contest particular characterizations, categorizations, or allegiances [49–51]. However, identity management is done not only through presenting but also through averting or concealing certain aspects of the self [52].

Social members can avoid stigmatization by concealing traits they find potentially discrediting [21]. This practice of concealment has both symbolic and material implications. In symbolic relations, it allows individuals to ‘pass’ (blend in) as ‘normal’ [21]. The material implications are particularly significant in neoliberal workplaces, where individuals are human resources evaluated by their competitiveness and market worth [53]. For instance, exposure of one’s mental ill health might lower their market worth as human resources that are ‘inferior’ or ‘defective’ (see the language of relationships above). This, in turn, might cause them to be deprived of opportunities and other resources (such as employment and promotion opportunities) in the workplace (see the discussion of discursive space above).

Identity management is practiced to avoid these potential risks. In other words, it is a strategy employed to navigate the mechanism of resource allocation [54, 55]. However, in conforming to the normative expectations of the workplace, identity management might perpetuate the established language of relationships. Persons with mental illness might still be denied access to resources and rejected by other members of society if exposed.

## Data and analytical approach

This study is based on findings from three initial surveys conducted by one of the co-authors and the City Mental Health Alliance Hong Kong (CMHA HK) in 2017, 2019, and 2020 [37, 56, 57]. Data was collected with over 1,500 respondents from the professional services industries in Hong Kong and focused on the mental health needs of employees. Participants were primarily from the legal, financial, and consulting sectors. All participants in the 2019 and 2020 surveys were invited to provide their contact information to take part in a post-survey interview. Participants who volunteered for an interview were contacted by our research team via email.

The surveys showed three main obstacles to mental health in the workplace. These were: limited mental health awareness, insufficient company support, and lack of trust. Findings were used to guide our initial design of the interview questions. The interviews were semi-structured, with the questions focusing on participants’ personal experiences of the mental health environment, training and communication in the workplace. The present paper is based on the corpus of interviews collected (Table 1).

Our corpus comprises of 23 in-depth interviews, with a demographic distribution as follows:

**Table 1:** Demographic distribution of interview participants

Gender	Male		Female
	8		15
Socio-cultural background	Local		Non-local
	13		10
Language	Cantonese	English	Mandarin
	10	13	0

‘Locals’ were defined as individuals who were born in Hong Kong or have lived in Hong Kong most of their life before reaching adulthood. ‘Non-locals’ were defined as individuals who were born outside Hong Kong and lived outside Hong Kong for most of their life before moving there. All participants had been in their occupation for at least three years at the time of the interview. Participants were offered the choice to have the interviews conducted in Cantonese, English, or Mandarin. These languages were suggested by the research team with reference to Hong Kong’s policy of biliteracy (Chinese and English) and trilingualism (Cantonese, English and Mandarin).

The interview recordings were transcribed and cross-checked by three research team members. All three transcribers were English-Chinese bilinguals. To allow the data set wider compatibility with different analytical methods, the team followed a detailed transcription notation [58]. While conversation analysis is not used in this paper, some notations were retained in the examples to provide a more comprehensive view of interviewees’ speech, use of language and the interview contexts. All personal identifiers have been anonymized in the transcripts. In the extracts below, participants are labelled ‘P’ and interviewers are labelled ‘I’. Interviews conducted in Cantonese were first transcribed in Chinese text, then translated into English. The transcriptions were verified by the three research team members to check for content and translation accuracy.

The analysis of the interviews is conducted using a theme-oriented discourse analytic approach [59]. It is guided by the assumption that “language does not just reflect or express intentions or decisions (the representational role of language); it makes them (the constitutive role of language)” [59] (p632). The interviews are analysed with the reflexive awareness on two levels. First, the recorded accounts are meta-discursive reflections of lived experiences – that is, the interviews are discourses about interactions in the workplace instead of the interactions in themselves [60]. Second, the interviews are co-constructive events between the interviewers and the participants instead of a one-way extraction of information [61].

The focal theme of ‘workplace stigma’ was generated from repeated listening of the recordings by the first author and two other research assistants on the team [59]. Initial mapping of the data showed diverse accounts on stigma-related experiences, such as acts of concealment, worries of jeopardized career, anticipations of a biased treatment, and normative pressures of the society. These noted accounts were coded, reviewed and discussed between the authors. Further analysis by the first author noticed the multi-scalar relations between the different types of reported experiences. This led to the generation of the three thematic layers: immediate contacts, organizational practices, and societal ideologies. These themes were discussed and refined between the three authors to ensure a good fit with the data.

Our theoretical framework – stigma as a language of relationship, the workplace as discursive space, and identity management as discursive practice – was developed based on these analytical findings. Theoretical insights were then combined into the Butterfly Model of Mental Health Stigma in Organizational Settings (see Discussion). The analysis that follows demonstrates how mental health stigma is produced in the workplace. We map the discursive practices circulating across the three layers. In particular, the data extracts show how identity management as a discursive practice is determined by the anticipated risks of alienation (i.e., being segregated from the ‘normal’ members of the workplace). This demonstrates how stigma functions as a regulative mechanism, and how this mechanism is discursively produced and perpetuated in the workplace.

## Analysis: the discursive structure of mental health stigma in the workplace

The analysis demonstrates how the discursive practices circulating across immediate contacts, organizational practices, and societal ideologies are entangled with identity management in the workplace. Immediate contacts refer to mental health stigma experienced in direct interactions between individual members of the workplace (Extract 1). Organizational practices is stigma experienced in the workplace’s structural organization (Extract 2). Societal ideologies correlate with stigma experienced in local traditional and global corporate value systems in the workplace (Extract 3 & 4). Participants’ accounts show that identity management is practiced in response to potential risks of alienation permeating the three layers. By examining how these risks are discursively reinforced across the layers, we demonstrate how stigma is perpetuated as a regulative mechanism in the workplace. We begin our analysis with immediate contacts, the layer closest to the stigmatized individual.

### Immediate encounters

#### Extract 1: Risks and distrust

*Background: The participant is a female executive of a multinational brand. She was diagnosed with bipolar disorder and experienced 2 years of prolonged stress. She has been on regular medication. The participant moved to Hong Kong 18 years ago. The interview was conducted in English. In the turns preceding this extract, the participant noted that work is her main source of stress.*

**Table:** 

1.	I:	oh so su- that means that often times even when you also have to do work as well
2.	P:	= oh yeah yeah I’ve lost complete ((inaudible)) of my work-life balance there’s no such thing at the moment.h and you know I’m to blame because I’m I- very fortunate I work in an environment where I s- if I say to my boss (.) um I say to her yesterday I’m not feeling good I want to go home. I’m going home I didn’t say I want to- I said going home, and she was like no problem. So I do it is a supportive environment but you know as open as this environment is (.) nobody here knows about my history so
3.	I:	@
4.	P:	= and that’s why
5.	I:	yeah
6.	P:	that’s why I’m so keen to talk
7.	I:	right
8.	P:	because I’m sure there must be many- many people like me um ((tongue clicking)) you know because this- the- this especially when you are.h (.) when you are in a position of leadership
9.	I:	right
10.	P:	there’s a risk. there’s a [big]
11.	I:	((murmuring)) [right]
12.	P:	big risk involved you know ((inaudible))
13.	I:	so you feel you couldn’t really disclose your issue with anyone else
14.	P:	= well I think I could um I mean I on personal level I have.h but on the work level (.) it’s a big risk and I haven’t want to take that risk up to now
15.	I:	Is it because of the associated stigma [or]
16.	P:	[yeah] absolutely (.) and I’m just about to get a promotion so
17.	I:	oh congratulations
18.	P:	@thank you@ thank you but you know it’s like hh
19.	I:	right
20.	P:	it’s not easy yeah
21.	I:	Um so e yu- you really feel that like there’s no like relationally that there’re no colleagues that you could really tell↑ or disclose to anyone↑
22.	P:	.h no because I don’t think people understand
23.	I:	okay
24.	P:	= and and then I think it becomes um it becomes an obstacle rather than anything else because.hh people don’t know how to treat you and [they]
25.	I:	[right]
26.	P:	= it’s kind of like you don’t want attention
27.	I:	right
28.	P:	so it just becomes s- becomes very complicated.hh I’ve got a headache is much easier to deal with for me↑ and for the person that is on the receiving end of that

In this extract, the participant presents a paradox in immediate contacts. On the one hand, the workplace is perceived to be ‘a supportive environment’ to members with mental illness (turn 2). On the other hand, the participant anticipates ‘a big big risk’ if her mental health condition were to be exposed (turns 10–12). To avoid the anticipated risk, the participant employs a strategy of identity management, namely, the somatization of her condition as ‘a headache’ (turn 28). This paradox raises two questions. First, what is the risk involved? Second, why is the risk anticipated despite the supportive environment?

The risk involved is hinted at in turns 8–12. As the participant notes, ‘when you are when you are in a position of leadership there’s a risk. There’s a big – big risk involved’. Here, the participant correlates the risk with her institutional position. The risk comes in the form of social awkwardness (turn 24) and unwanted attention (turn 26). However, noting particular reference to her institutional position, the situation of ‘people don’t know how to treat you’ might also imply the potential discrediting of the participant as a leader in the workplace (turn 24). That is, her team members might have difficulties working under her leadership, if her mental health condition is exposed, in line with evidence that workers with mental illnesses are often perceived as less competent or untrustworthy [11].

The problem of credibility could further impact an individual’s personal financial situation. As the participant notes later in the interview, ‘because there’s bigger price to pay in the corporate world because your job your payment on your paycheck and that makes all the difference’ (not included in Extract 1). This implies the possibility of salary reduction, or even problems with employment [10]. In immediate contacts, identity management is employed to avoid potential social awkwardness and unwanted attention. But these are not trivial matters of momentary encounters, as emphasized by the participants. There’s a ‘big big risk’ and ‘a bigger price’. For risks in one’s immediate contacts have deeper implications for the individual’s career prospects and livelihood.

We are not explicitly told why the supportive environment could not mitigate these risks. However, when the interviewer suggested stigma as a potential cause for the risks, the participant gave a strong affirmative response. This is shown by her overlapping response ‘yeah’, intense affirmation ‘absolutely’, and volunteered thoughts on the potential risks related to her promotion (turns 16–22). According to the participant, this need to carefully manage one’s own identity is specific to the workplace (turn 14). This is presented not as an experience of her own, but a normalized experience shared among ‘many many people like me’ (turn 8). In other words, these risks are seen as stigma taking place in the workplace. And they are linked to experiences on a more general level, instead of individual contacts. For this, we now turn to the layer of organizational practices.

### Organizational practices

#### Extract 2: Mental health training and performance evaluation

*Background: The participant is a female administrative staff member holding a temporary position in an international company in Hong Kong. She was diagnosed with bipolar disorder and has been experiencing frequent depressive episodes.*

**Table:** 

1.	I:	[好]明白(.)咁如果你UH經歷過呢啲係唔該(.)經歷過呢啲咁樣嘅: 即係精神壓力啦你會唔會選擇<同你嘅>同事啦上司啦或者係管理層談及呢啲問題架
		[right] got it (.) then if you have uh experienced these thank you (.) experienced these like: that is mental pressures would you choose to < talk to your > colleagues superiors or managers about these problem
2.	P:	唔會
		no
3.	I:	係完全唔會嘅
		it’s absolutely not
4.	P:	完全唔會
		absolutely not
5.	I:	係
		yes
6.	P:	因為我嗰班同事係 < 香港嘅 > base嘅
		because my colleagues are < Hong Kong > based
7.	I:	um hm
		um hm
8.	P:	咁: 係囉
		then: yes
9.	I:	Um hm
		um hm
10.	P:	同埋我唔sure佢哋嘅training夠唔夠囉就[算係嘅話]
		and also I am not sure if their training is enough or not ev[en if]
11.	I:	[um hm]喔: okay 係
		[um hm]oh: okay yes
12.	P:	但係就算training嘅話都要睇點train囉 [其實]-
		but even if for the training it also depends on how the training is carried out [actually]-
13.	I:	[um hm]
		[um hm]
14.	P:	-即係啊(.)即係加其實有啲難嘅因為你.h你覺得professionalism你一定要去到做到一個嘅standard but at the same time你有咩辦法係嘩即係okay understand (.) 啊professional啦你要達到個standard啦跟住跟住之後你有時啲symptom嘅嘢嗰個人係咪(.)真係懶.h定係佢真係個symptom
		= that is ah (.) that is actually it’s a bit difficult because you.h you think professionalism you have to reach achieve a standard but at the same time what can you do to whoa that is okay understand (.) ah to be professional you need to reach the standard then then you sometimes those symptoms if the person is (.) just lazy.h or is he/she in fact the symptom
15.	I:	UM
		um
16.	P:	嗰個人係真係concentrate唔到定係嗰個symptom
		-that person really cannot concentrate or is it the symptom
17.	I:	UM
		um
18.	P:	嗰個人cannot multitask係咪個(.)其實cannot multitask呢樣嘢(.)可圈可點啦吓-
		that person cannot multitask is it that (.) actually this thing about being unable to multitask is (.) ambiguous
[17 turns omitted]		
36.	P:	[嗰個nature] < and > 係囉
		[that nature] < and > yes
37.	I:	um hm
		um hm
38.	P:	同埋嗰個biased opinions啦
		and also those biased opinions

This extract suggests two related organizational practices that might have caused the general experience of stigma in the workplace. First is the ‘biased opinions’ in performance evaluation (turn 38). Second is the limited mental health training offered at work (turns 10–12). As the participant suggests, limited mental health training causes bias in performance evaluation. Identity management is employed to avoid the risk of being so evaluated. When asked whether she would discuss her mental health condition with others at work, the participant responds negatively (turn 2). The participant further confirms an intensified negation in repeating the interviewer’s check (turns 3–4). The questions for us here are these: how does limited mental health training lead to biased evaluation? How does this correlate with the risks observed in Extract 1?

The relationship between performance evaluation and mental health training is mediated by standards related to ‘professionalism’ (turn 14). Professionalism is defined by Fournier as an organizational practice that ‘work[s] to inculcate ‘appropriate’ work identities, conducts and practices’ [62, 63]. The participant points to two particular qualities required to be of professional standard: *concentration* (turn 16) and *multitasking* (turn 18). While these qualities might be agreeable by themselves, they could be problematic when incorporated into employee evaluation practice. This is especially the case when evaluators are not sufficiently well-trained in handling mental health issues in the workplace. As the participant notes, without sufficient training, it is difficult to differentiate between a person showing symptoms of their mental health condition and a person lacking professional qualities (turns 10–18). Symptoms of mental ill health might also be labelled as personality flaws such as being ‘lazy’ (turn 14) [64]. In other words, insufficient mental health training prevents accurate judgments being made in performance evaluations.

These ‘biased opinions’ (turn 38) place individuals with mental illnesses in an unfavourable position in performance evaluation. This aligns with the anticipated risks discussed in Extract 1. The lack of mental health training in the workplace has given rise to ‘biased opinions,’ which prevents evaluative practices from drawing accurate judgments (Extract 2). The potential career consequences have led to the anticipation of risks in disclosure (Extract 1). Individuals employ identity management to ‘pass’ in the organizational practice of evaluation and, by so doing, avoid potential loss of career opportunities. Disclosure in immediate contacts might bring consequences from organizational practices. Reciprocally, the anticipation of these consequences led to refraining from disclosure in immediate contacts. This shows how stigma permeates and regulates interactions in these two layers.

However, it is necessary to ask whether the provision of more mental health training by itself would reduce stigma in the workplace? Training is one suggested intervention increasingly recommended to tackle gaps in mental health knowledge [65]. Yet, it is doubtful whether the mere increase in quantity might solve the problem. As the participant criticizes later in the interview, the mental health training programmes that she is aware of are either medicalizing mental health or presenting it in an overly emotional manner (163 turns after Extract 2). The debates around mental health training also abound in the literature [66, 67]. However, here we note that Extract 2 still points to two influences from beyond the immediate workplace. First is the local culture of Hong Kong (turn 6) and second is the corporate culture in which professionalism is produced (turn 14). This brings us to the layer of societal ideologies.

### Societal ideologies

#### Extract 3: Chinese cultural values

*Background: The participant is a female translator working at an independent body in Hong Kong. Her employment started in late 2017 after returning to Hong Kong about a year prior to the time of this interview. She resigned from the job after a few months due to a relapse into depression. She was unemployed at the time of the interview.*

**Table:** 

1	I:	okay(.)好(.)好(.)咁uh: 我哋都想知道你認為UH > 有幾大嘅 < 程度同埋喺咩方面呢啲 < 職場嘅精神壓力 > 同成個社會氣氛係有關嘅
		okay (.) right (.) right then uh: we also want to know you thoughts uh > to what < extent and in what way this < mental pressure > in the workplace is related to the social atmosphere
2	P:	都幾大: 關係嘅
		(1.0) quite significantly: related
3	I:	um
		um
4	P:	咁(.) uh成個社會氣氛其實(.) uh (.)一方(.)咁(.)社會上都係我哋都係華人為主啦跟住咁你我哋嘅工作公司都係華人為主啦(.)就即使係嗰啲: 所謂外國人做: CEO咁其實都唔會話好大差別 > 因為 < 你主要都係(.)華人嘅員工啦(.)咁佢哋個個觀念都係會相似嘅都係會uh: 容易覺得uh > 精神健康 < 呢方面係你自己應該要搞掂嘅uh你搞唔掂就係你嘅問題(.)uh: 同埋都會uh(.)就好似頭先講 > 即係 < < 上面對下屬 > 上司對下屬嗰種嘅: 關係(.)會uh: 容易啲畀人有壓力囉
		then (.) uh the wider social atmosphere actually (.) uh (.) for one thing (.) then (.) socially we are Chinese dominant and then your our job companies are also Chinese dominant (.) even with those: so-called foreigners as: CEO that actually it wouldn't be of much difference > because < you have mainly (.) Chinese employees (.) well their concept would still be similar would be uh: easily thinking uh of things related to > mental health < as you personally should handle it well uh if you cannot handle it well then that is your problem (.) uh: and also it would uh (.) like what I have said > that is < < the top to the subordinates > the superiors to the subordinates that kind of: relationship (.) would uh: easily put pressure on people
5	I:	um (.)而你覺得最:: 主要嘅原因就係因為我哋係華人社會有呢種文化
		um (.) and you think the most:: important reason is that because of this culture we have in our Chinese society
6	P:	係uh都幾根深蒂固嘅
		it is uh quite deep-rooted

Hong Kong is often described as a city where East meets West. As suggested by the participant, international corporate offices in Hong Kong might employ a foreigner as CEO, leading a team consisting predominantly of Chinese employees (turn 4).

The participant argues, however, that the workplace is strongly influenced by ‘deep-rooted’ Chinese cultural values (turns 4–6). It is clear that there are many cultural influences on mental health [68, 69] but we note here that the participant points particularly to the idea that mental health is one’s personal responsibility and ‘if you cannot handle it well then that is your problem’ (turn 4). Such an emphasis on self-responsibility can be argued to be echoed in the Confucian ethics of self-restraint and self-cultivation, [43] and is related to the socially assumed correlation between mental ill health and personal moral failure. This relates back to the practice of evaluation in the workplace (Extract 2). However, the focus here is not on the individual’s productive competence but in their moral conduct. Given this, it could be argued that identity management is employed not only to ‘pass’ as a competent employee but as a person of moral standing.

It is worth noting that the label ‘Chinese’ is invoked by the participant as an ethnic-cultural (‘華人 *waa4jan4*’) instead of national (‘中國人 *zung1gwok3jan4*’) category (turn 4). This could be read in connection with the earlier remark ‘because my colleagues are Hong Kong based’ (Extract 3, turn 6). It might not be the geographical location that concerns the participant in Extract 3. Rather, it might be the dominant cultural code. Here therefore, it is possible that the value of self-responsibility could be brought into the workplace by other colleagues immersed in this cultural code, thus reinforcing stigma against persons with mental health conditions at work. In such cases, a person with mental illness would be perceived as a moral deviant. However, a sole focus on the influence of Chinese cultural values on mental health stigma would be an omission of the importance of Hong Kong’s social environment and multi-national working environment.

#### Extract 4: Global corporate values

*Background: The participant is a female human resources manager at an international law firm in Hong Kong. She left her previous job due to stress and pressure and was away from the labour market for several years before taking up the position at her current firm.*

**Table:** 

1	I:	so um do you feel think that there are some persistent um do you think that for in society that there’re some persistent or recently emerging um mental health challenges↑
2	P:	you’re going back to society not workplace
3	I:	um (.) well workplace and society yeah like (.) both it can happen to both levels
4	P:	um: I think it goes back to the taboo the- the stigma of talking about mental health↑ um: if it exist outside of work then definitely it- it exist in the workplace↑ and: I think in a (.) in the in our kind l- workplace people tend to be first of all it’s the level of (.) um (.) we are the sort of firm that we are the probably the the top layer of all the international law firms↑ and so we hire um the the the probably the best professionals that we can get on our staff↑ so there’s a certain expectation from um the sort of people who work with us to be high performers high flyers and they probably were the top students when they were at school when they were at university and so when they are here they are expected to be performing (.) at the top of their game. and I think that that sometimes (.) um: become an issue in itself↑ you you’re not gonna admit to failure you’re not gonna admit to weaknesses
5	I:	(right)
6	P:	easily↑ and if that’s coupled with the general (.) stigmatization of talking about mental health (.) in the wider context you know in your community at home or amongst your friends because your friends are probably also the grade A students back then↑ you’re not gonna be able to open-up and speak more easily. about (.) any challenges that you feel

This extract begins with the interviewer’s question on persistent mental health challenges in society (turn 1). In identifying these challenges, the participant points to ‘the stigma of talking about mental health’ (turn 4). However, her subsequent elaboration alludes more specifically to the corporate value of elitism. Elitism is defined by Jaworski and Thurlow as ‘a person’s orientation or making a claim to exclusivity, superiority, and/or distinctiveness on the grounds of status, knowledge […] or any other quality warranting the speaker/author to take a higher […] standing in relation to anther subject (individual or group)’ [70] (p196). In other words, it is a value emphasizing excellence and competition. Identity management is employed to prevent oneself from being perceived as falling short of these corporate expectations. This is explained in length by the participant (turn 4).

Elitism is practiced both by the corporation and the individual. The corporation hires only ‘the best professionals that [they] can get on [their staff]’ (turn 4). Recruited members are expected ‘to be high performers high flyers’ (turn 4). They are expected to be ‘at the top of their game’ (turn 4). Talking about mental health at work would be judged as an admission of ‘failure’ and ‘weakness’ (turn 4). At the same time, the individual constantly places themselves under intense competition with others to be the best (elitism). Admitting to ‘failure’ and ‘weakness’ would also mean admitting defeat in this competition, especially among peers (turn 6). This includes not only colleagues at work, but also one’s ‘community at home’ and one’s ‘friends’ (turn 6). It could be argued that identity management is employed to demonstrate one’s quality beyond the mere competence in completing the tasks. As such, it is practiced in sustaining one’s ‘superiority’ and ‘distinctiveness’ over others at work and in society.

Such competition may be continuous and related to a competitive culture permeating the corporate labour market. As Harvey suggests in his critique of neoliberalism, individuals are evaluated by corporations ‘in terms of entrepreneurial virtues or personal failings’ [53] (p65). Correspondingly, individuals are expected to continuously strive for success and excellence [71]. Only by doing so can they maintain their market value as human resources. This, again, ties back to the performance evaluation in organizational practices (Extract 2). As the values of elitism and neoliberalism enter the workplace, they become part of professional work ethics. Consequently, talking about mental health might be ‘taboo’ (turn 4). Breaching this taboo might be taken as reflecting one’s failure to uphold professional work ethics, or even one’s personal flaws e.g., laziness [64]. In turn, this creates the potential risks in losing career opportunities, which might bring negative impact on other aspects of one’s personal life (Extract 1).

In combination, Extracts 3 and 4 suggest societal ideologies that might be incorporated in the organizational practice of performance evaluation. Workplaces in Hong Kong are, on the one hand, influenced by the Chinese cultural value of self-responsibility. On the other, they are impacted by the global corporate values of elitism and neoliberalism. It is crucial to recognize this connection between societal ideologies and the internal evaluative practices of organizations. As the participant in Extract 4 suggests, ‘if [stigma] exist [sic] outside of work then definitely it it exist [sic] in the workplace’ (turn 4). The risks anticipated by individuals in immediate contact cannot be understood solely via practices within the organization and such risks are often related to values circulating in wider society. Stigma is perpetuated in this circulation of discursive practices reinforcing, and averting, these risks.

## Discussion

The above analysis illustrates how stigma is discursively produced, and how it is perpetuated as a regulative mechanism in the workplace. As participants and resources enter the workplace, they bring with them values circulating in society. In Extracts 3 and 4, we can see the combination of Chinese cultural values (e.g., self-responsibility) and global corporate values (e.g., excellence and competition). These values feed into the organizational practices of performance evaluation. With limited mental health training, evaluators have difficulties in distinguishing symptoms of mental illness from personal qualities of the individual (Extract 2). This potential misjudgement in evaluation creates the anticipation of risk in disclosing one’s mental health status to immediate contacts (Extract 1). This dynamic circulation of discursive practices creates a regulative mechanism. By inducing the risks of alienation, stigma regulates interpersonal relationships (how the ‘deviant’ should interact with the ‘normal’) and resources allocation (who should be promoted or employed).

**Fig. 1:**
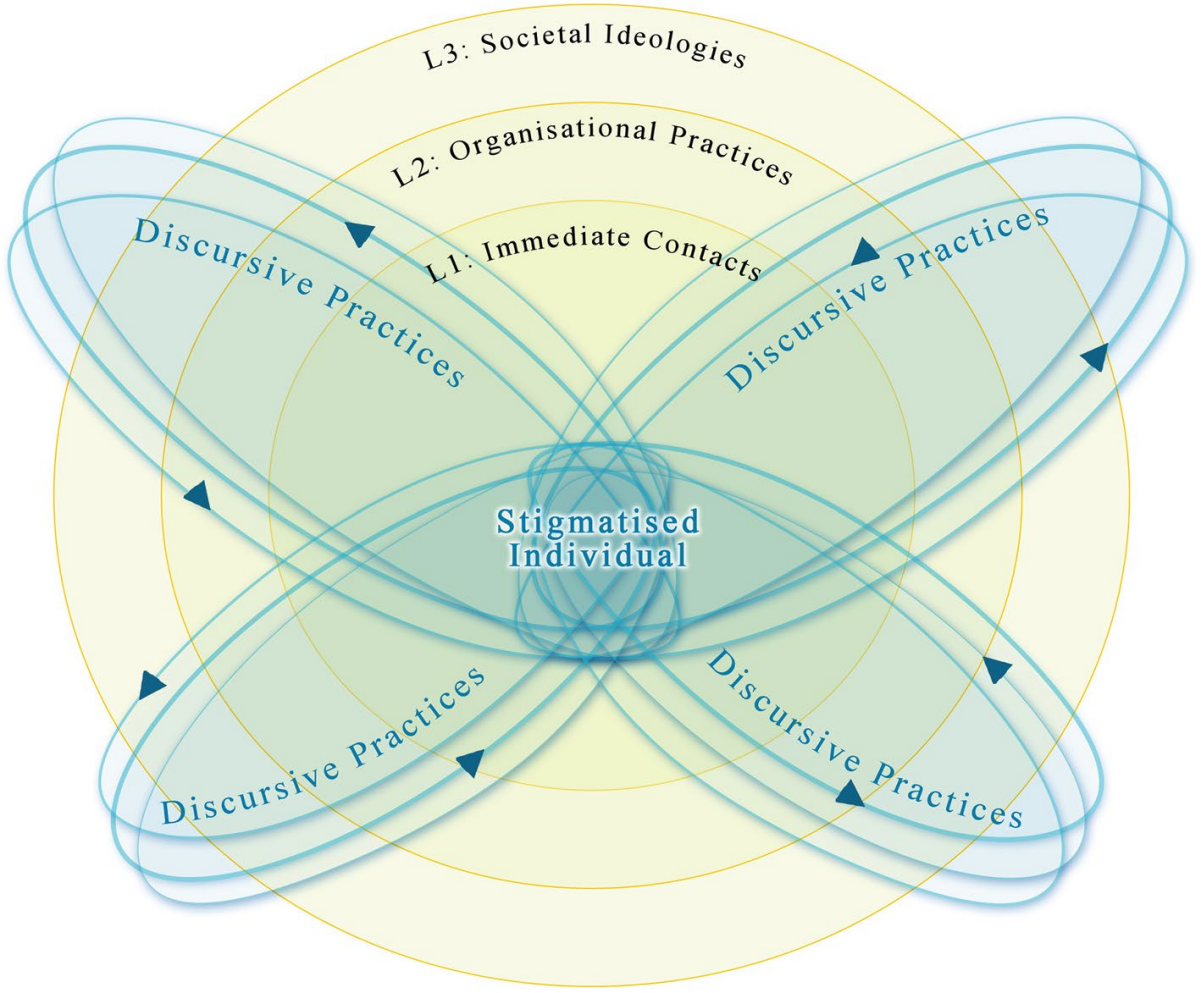
The butterfly model of mental health stigma in organizational settings

Based on these findings, we offer the Butterfly Model of Mental Health Stigma in Organizational Settings (Fig. 1) to map the connections between the stigmatized individual caught in a language of relationships, the workplace as discursive space, and identity management as discursive practice. Recent sociological research has recognized the multi-layered production of mental health stigma, but it remains a challenge to map the interconnections between “discourses operating at each level [that] influence people experiencing mental illness in workplace settings” [16] (p959). Our Butterfly Model offers a sociolinguistic approach to this problem. As Heller puts it, sociolinguistics is particularly well-suited “to capture the processes of construction of category and subjectivity as they unfold and to identity the interactional means by which inequality happens” [14] (p11). The Model maps the production of stigma in discursive practices circulating across multiple sites of interaction, and the symbolic and material order of alienation formed in this circulation of discursive practices.

First, the ripple in the Model (L1, L2 and L3) depicts a multi-layered *discursive space*. Our analysis presents the production of stigma across a three-layered discursive space: immediate contacts, organizational practices, and societal ideologies (e.g., face-to-face meetings, performance evaluations, and social judgments). This multi-layered space is formed by discursive practices performed at multiple sites. These sites could be within or beyond the organization, for instance, the office space (Extract 1), one’s community at home (Extract 4), and the media [11]. This is shown by the loci of discursive practices circulating not in a single orbit but in multiple loops. In combination, the ripples and the wings convey the first property of stigma as a regulative mechanism: it is multi-layered and multi-sited.

Second, the ‘spreading wings’ depict the *discursive practices* circulating across the three layers. While our analysis focuses on the practice of identity management, it is only one type of discursive practice involved in the production of mental health stigma in the workplace. For this reason, we use the general term ‘discursive practice’ instead of the specific type ‘identity management’ in the Model. As our analysis demonstrates, stigma is not formed in the mere combination of discursive practices taking place at each level [16] (p959). Rather, interactions between identity management and other discursive practices (e.g., ‘biased opinions’ in performance evaluation and moral discrediting in social judgment) develop across the multiple layers. The dynamic nature of the discursive practices is conveyed by the arrows on the loci. In combination, the wings and the arrows depict the second property of the regulative mechanism: it is enacted in dynamic discursive practices.

Third, the body of the butterfly depicts the stigmatized individual. As reflected in the extracts, stigma is a *language of relationships* that postulates the distinction between the ‘deviant’ and the ‘normal’. This language incorporates values of social judgment (e.g., Confucianism, elitism, and neoliberalism; see Extracts 3 and 4). Permeating the organizational practices of evaluation, it influences the gatekeeping of resources (e.g., promotion and employment opportunities). Individuals with mental illnesses might be judged as falling short of the professional standard and might thus lose their career opportunities. These potential risks regulate how individuals interact with other members in the workplace (e.g., they may conceal their mental health condition). This nexus constituting the butterfly’s body depicts the third property of the regulative mechanism: it influences both interpersonal relationships and resource allocation in the workplace.

The Butterfly Model highlights three properties of mental health stigma as a regulative mechanism as discussed above. The Model offers a sufficiently restrictive guide for showing how stigma is formed in discursive practices circulating across the three layers, and how it regulates interpersonal relationships and resource allocation in a particular organizational setting. At the same time, the Model leaves enough room to accommodate contextual variations. We summarize six practical guiding questions:

- What are the indicative discursive practices observed in immediate contacts?
- How might organizational practices have triggered those practices in immediate contacts?
- How have societal ideologies informed these organizational practices?
- How do these discursive practices connect the different layers?
- How are symbolic relations and material resources allocated in the discursive space formed?
- What language of relationships is employed in organizations to rationalize these allocations?

Our goal is not to develop a programmatic agenda for mental health stigma research in sociolinguistics. Rather, we offer the Butterfly Model as a conceptual tool to identify plausible directions for analysis in future research. It offers a perspective into the multi-layered and multi-sited discursive space of organizations. The processes of stigmatization are driven by dynamic discursive practices circulating across the layers and sites. The stigmatized individual is produced through these processes in a language of relationships, regulating symbolic relations, and material resources in organizations.

## Conclusion

The COVID-19 pandemic has come to an end [72]. However, the challenge of its co-pandemic, that is, the global deterioration of mental health remains. Stigma stands as one persistent obstacle to tackling mental ill health. In this paper, we offer the Butterfly Model of Stigma in Organizational Setting to facilitate the analysis of how mental health stigma is discursively produced, and how it functions as a regulative mechanism in the workplace. The theoretical insights are illustrated through a meta-discursive analysis of workplace experience in Hong Kong. The analysis demonstrates how discursive practices, such as identity management, interact with other practices across immediate contacts, organizational practices, and societal ideologies. These layers are tied into producing a language of relationships, in which individuals with mental illnesses are categorized as ‘deviants’. In rationalizing the alienation of these individuals, this kind of language creates potential risks for lost career opportunities. Stigma perpetuates and manifests in silence, that is refusal to discuss mental health openly. The Butterfly Model provides a conceptual tool for analyzing and potentially acting on mental health stigma in the workplace.

## Transcription notations

**Table:** 

(.)	Brief interval
Word-	Cut-off
(0.0)	Elapsed time
word	Emphasis
↑	Heightened pitch
.hh	Inbreath
@	Laughter
=	No break or gap
hh	Outbreath
[]	Overlap
:	Prolongation
< >	Slowed down
> <	Speed up
(())	Transcriber’s remarks

## Data Availability

The datasets generated and/or analysed during the current study are not publicly available due to the need to protect individual privacy of the participants but are available from the corresponding author on reasonable request.
